# Pressure changes in the endotracheal tube cuff in otorhinolaryngologic surgery: a prospective observational study

**DOI:** 10.3389/fmed.2023.1161566

**Published:** 2023-06-05

**Authors:** Sujung Park, Young In Kwon, Hyun Joo Kim

**Affiliations:** Department of Anesthesiology and Pain Medicine, Anesthesia and Pain Research Institute, Yonsei University College of Medicine, Seoul, Republic of Korea

**Keywords:** cuffed tube, endotracheal tube, extubation, intubation, otorhinolaryngologic surgery, pressure changes, cuff pressure, continuous monitoring

## Abstract

**Objective:**

Inflation of the endotracheal tube cuff is needed for providing ventilation. Cuff pressure should be maintained inside the appropriate range to prevent critical airway complications. The purpose of this study is to evaluate the pressure changes in the endotracheal tube cuff during otorhinolaryngologic surgery.

**Design and method:**

This single-center observational study was conducted at Severance Hospital in Korea between April 2020 and November 2020. Patients aged >20 years scheduled to undergo otorhinolaryngological surgical procedures were enrolled. Patients undergoing planned tracheostomy and those who were slated for uncuffed endotracheal tube use were excluded. Intubation was performed after the induction of general anesthesia. A pressure transducer was connected to the pilot balloon of the endotracheal tube, and cuff pressure was continuously monitored until extubation. If the cuff pressure was not appropriate for more than 5 min, it was adjusted to the appropriate range by injecting or removing air. The percentage of time for which the cuff pressure remained within the appropriate range was calculated and defined as the time in the therapeutic range (TTR). The presumed cause for the rise or fall in cuff pressure was identified.

**Results:**

In total 199 patients, alterations in cuff pressure outside the appropriate range occurred in 191 patients (96.0%). The mean TTR was 79.7% (SD 25.0%), and head and neck surgery had the lowest mean TTR of 69.0% compared to ear and nose surgeries (94.2 and 82.1%, respectively). Sixty-eight patients (34.2%) demonstrated inadequate endotracheal tube cuff pressure for more than 20% of the total anesthesia time. Twenty-six patients (13.1%) demonstrated optimal endotracheal tube cuff pressure for less than 50% of the total anesthesia time. The causative factors inducing inappropriate cuff pressure were found to vary, including positional changes, surgical procedure, anatomical manipulation, and anesthetic procedure.

**Conclusion:**

In otorhinolaryngologic surgery, cuff pressure increased or decreased outside the appropriate range due to various factors. Therefore, we suggest close continuous monitoring of cuff pressure during anesthesia for otorhinolaryngologic surgery.

**Clinical trial registration:**

clinicaltrials.gov, identifier NCT03938493.

## 1. Introduction

Endotracheal intubation is a common practice for securing and maintaining the airway in general anesthesia. The optimal endotracheal tube (ETT) cuff pressure is 20 to 30 cmH_2_O (1). High pressure in the ETT cuff produces ischemic changes in the tracheal mucosa and compresses the structures around the cuff (2), which can result in critical complications, such as vocal cord paralysis, recurrent laryngeal nerve injury, tracheal stenosis, and tracheal rupture (3). Conversely, insufficient cuff pressure can lead to a significant leak around the ETT or microaspiration (4). Thus, many anesthesiologists recommend that the ETT cuff pressure should be adjusted to an appropriate level (5–7).

In routine practice, the ETT cuff is usually checked once by palpating the pilot balloon or using a manometer immediately after intubation (8). However, the ETT cuff pressure undergoes subsequent changes based on various factors, such as airway pressure and patient positioning during surgery (2, 9). Abnormal ETT cuff pressure during surgery reportedly leads to clinically significant adverse effects such as postoperative sore throat, hoarseness, cough, tracheal injury, and silent aspiration (10, 11). Therefore, continuous monitoring of the ETT cuff pressure during surgery after the induction of anesthesia is crucial.

Otorhinolaryngologic surgery requires changing the posture of the patient’s head and neck. In the flexed position, the pressure of the tube to the anterior of the laryngopharynx was reported to be higher than that in the neutral position (12). However, in the extended position, the mucosal pressures in the anterior and posterior cuff were demonstrated to increase (13). Therefore, in otorhinolaryngologic surgery, the cuff pressure may be inappropriate depending on the patient’s posture, and since the surgical site is proximate to the airway, there is a risk that the cuff pressure may change due to factors other than postural changes. However, to the best of our knowledge, no study has been conducted to continuously observe the changes in the ETT cuff pressure during anesthesia in otorhinolaryngologic surgery, including in ear, nose, and head and neck surgeries.

In this study, we aimed to evaluate the pressure changes in the ETT cuff during otorhinolaryngologic surgery. We hypothesized that the cuff pressure during otorhinolaryngologic surgery would vary outside the optimal range. Moreover, we aimed to identify the factors that induce inappropriate ETT cuff pressure.

## 2. Materials and methods

This prospective, single-center, observational study was conducted at the Severance Hospital, Yonsei University Health System, Seoul, Republic of Korea. This study was approved by the University’s Institutional Review Board (IRB # 4-2019-0100), and written informed consent was obtained from all the individuals participating in the trial. The trial was registered prior to patient enrollment at clinicaltrials.gov (NCT03938493, Principal investigator: HK, Date of registration: 6 May 2019).

Patients aged more than 20 years scheduled to undergo otorhinolaryngologic surgical procedures from April 2020 to November 2020 were enrolled in this study. Patients undergoing tracheostomy during the procedure and cases where uncuffed endotracheal tubes had to be used were excluded.

Each patient was monitored using electrocardiography, non-invasive blood pressure measurements, and pulse oximetry before induction. General anesthesia was induced by following the routine protocol of the institute that included preoxygenation with 100% oxygen; induction with propofol 2–3 mg kg^–1^, remifentanil 0.05–0.30 μg kg^–1^ min^–1^, and rocuronium 0.4–0.6 mg kg^–1^; and tracheal intubation with a cuffed tracheal tube of an appropriate size. After tracheal intubation with a cuffed ETT, the cuff was inflated by the anesthesia provider using air with a 10 ml syringe, and initial mechanical ventilation was commenced in a volume-targeted pressure-controlled mode set to deliver a tidal volume of 6 mL kg^–1^, PEEP 5 cmH_2_O, and 0.5 inspired oxygen fraction. A transducer (TruWave Disposable Pressure Transducers PX260, Edward Lifesciences, Irvine, CA, USA, Supplementary Figure 1) from a standard invasive pressure monitoring device that is routinely used to measure arterial or central venous pressure was attached to the pilot balloon of the ETT (9). The cuff pressure of the ETT was continuously recorded every 10 s, from tracheal intubation to just before extubation after the surgery. If the cuff pressure deviated from the normal range (15–22 mmHg) for more than 5 min, air was added or removed to maintain the cuff pressure within the appropriate range. The presumed cause for the rise or fall of the cuff pressure outside the optimal range was identified and recorded. After discussing the cause with the surgeon, efforts were made to maintain optimal cuff pressure by adjusting the pressure on the airway in the surgical field.

Following the popularization of the concept of time in the therapeutic range (TTR) for reporting anticoagulation management (14), TTR represented the percentage of time in which the cuff pressure remained in the appropriate range of 15 to 22 mmHg across the total anesthesia time in this study. TTR was calculated from the point at which the first cuff pressure ranged between 15 and 22 mmHg to remove the abnormal cuff pressure before connecting the transducer to the pilot balloon. A smoothing method was used to eliminate the extreme outliers in cuff pressure values, such as when the device was disconnected or dropped or when the value changed for a very short period. Supplementary Figure 2 shows an example of the application of smoothing methods.

We calculated the minimum sample size required to identify cases where the ETT cuff pressure exceeded the normal range in patients undergoing otorhinolaryngologic surgery. The change in the ETT cuff pressure was reported to be approximately 17% (15); thus, we aimed to recruit approximately 200 patients.

Continuous variables are reported as the mean ± standard deviation, and categorical variables are reported as numbers (percentages). Continuous variables were analyzed using Student’s *t*-test or Mann–Whitney U test, as appropriate. Categorical variables were analyzed using the chi-square test or Fisher’s exact test. All analyses were performed using R package version 4.1.0 statistical software (The R Foundation for Statistical Computing, Vienna, Austria).^1^ Statistical significance was defined as a two-sided *p*-value < 0.05.

## 3. Results

The final analysis included 199 patients. Table 1 presents the patients’ characteristics. In cases where more than two of the otorhinolaryngologic parts were involved, the case was classified as the main type of operation. Based on the operator’s request, a 6.0-sized ETT was used during laryngomicrosurgery and pulsed dye laser surgery.

**TABLE 1:** Patient characteristics.

	*N* = 199
Age, years	52.2 ± 16.8
Sex (Female)	94 (47.2)
Weight, kg	65.6 ± 12.2
Height, cm	165.1 ± 8.7
Body mass index, kg/m^2^	24.2 ± 4.8
**Endotracheal tube size, ID (mm)**	
6	21 (10.6)
6.5	92 (46.2)
7	2 (1.0)
7.5	84 (42.2)
**Endotracheal tube type**	
Wire	124 (62.3)
Plain	68 (34.2)
RAE	7 (3.5)
**American Society of Anesthesiologists class**	
I	79 (39.7)
II	80 (40.2)
III	40 (20.1)
Operation time, min	112.0 ± 95.6
Anesthesia time, min	148.1 ± 103.3
**Types of surgeries**	
Nose	95 (47.7)
Ear	35 (17.6)
Head and neck	69 (34.7)

Data are presented as the mean ± standard deviation or number (percentage). ID, internal diameter; RAE, Ring-Adair-Elwyn.

**FIGURE 1:**
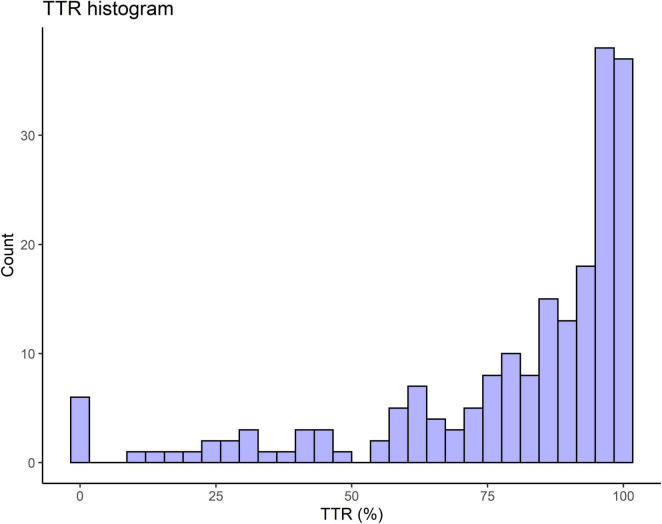
Histogram showing the frequencies of time in therapeutic range (TTR) in otorhinolaryngologic surgery. TTR represents the percentage of time in which the cuff pressure remains in the appropriate range against the total anesthesia time.

Alterations in the ETT cuff pressure occurred during the otorhinolaryngologic surgical procedures. Only 8 patients (4%) were observed to have a TTR of 100%, which indicated appropriate maintenance of cuff pressure within the appropriate range throughout the anesthesia time (Figure 1 and Table 2). The mean TTR was 79.7% (SD 25.0%). There were 68 patients (34.2%) with a TTR < 80% who had inadequate cuff pressure for more than 20% of the total anesthesia time. Moreover, 26 patients (13.1%) had cuff pressures that varied and were out of the appropriate range for more than half of the total anesthesia time.

**TABLE 2:** Time in therapeutic range (TTR) according to the operation type.

	Nose (*n* = 95)	Ear (*n* = 35)	Head and neck (*n* = 69)	Total (*n* = 199)
TTR (%)	82.1 ± 22.2	94.2 ± 7.9	69.0 ± 29.6	79.7 ± 25.0
TTR = 100%	6 (6.3)	2 (5.7)	0 (0.0)	8 (4.0)
TTR < 90%	46 (48.4)	7 (20.0)	48 (69.6)	101 (50.8)
TTR < 80%	29 (30.5)	3 (8.6)	36 (52.2)	68 (34.2)
TTR < 70%	20 (21.1)	1 (2.9)	26 (37.7)	47 (23.6)
TTR < 60%	14 (14.7)	0 (0.0)	19 (27.5)	33 (16.6)
TTR < 50%	9 (9.5)	0 (0.0)	17 (24.6)	26 (13.1)

Data are presented as the mean ± SD or number (percentage). SD, standard deviation; TTR, time in therapeutic range. TTR represents the percentage of time in which the cuff pressure remains in the appropriate range of 15 to 22 mmHg across the total anesthesia time.

**FIGURE 2:**
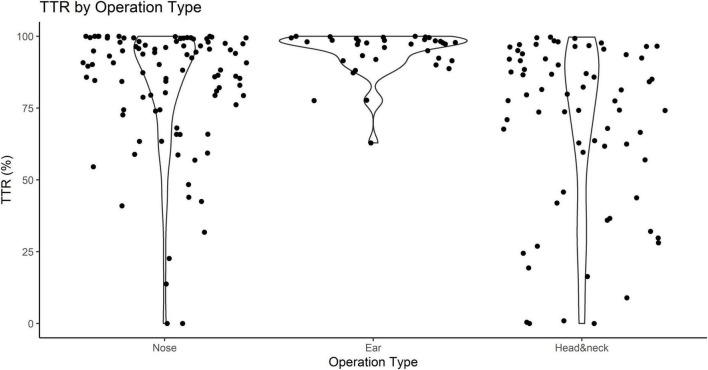
Time in therapeutic range (TTR) according to the surgical site classification of otorhinolaryngologic surgery. TTR represents the percentage of time in which the cuff pressure remains in the appropriate range against the total anesthesia time.

Although the clinician deflated and inflated the cuff to properly adjust the pressure, stabilizing it in the appropriate range throughout the surgery was challenging as the situation in the operating field changes from moment to moment. The cuff pressure in ear surgery was best maintained within the optimal range and had a mean TTR of 94.2%. Meanwhile, head and neck surgery showed the lowest mean TTR of 69.0%, indicating that the cuff pressure changed more frequently and was above or below the optimal range more often (Figure 2).

Table 3 presents the factors inducing inappropriate intracuff pressures during surgery. Neck flexion, neck rotation, chin lift, and the insertion or removal of the shoulder roll induced cuff pressure changes, and these factors were classified as positioning. Factors such as the insertion of a suspension laryngoscope, removal of a suspension laryngoscope, oral suction, insertion of tonsillar forceps, removal of an army instrument, start of the operation, and draping were identified and categorized as surgical procedures. Anatomic manipulation of the patients, including external laryngeal pressure, pulling the tongue, and nasal packing, was found to change cuff pressure. Insertion of the temperature probe into the esophagus or hiccup during the surgery was shown to cause cuff pressure changes and was defined as an anesthetic procedure. Other than the removal of instruments, all of the aforementioned causative factors led to an increase in the ETT cuff pressure.

**TABLE 3:** Causative factors that induce cuff pressure changes outside the optimal range during anesthesia.

Factors	Nose (*n* = 95)	Ear (*n* = 35)	Head and neck (*n* = 69)	Total (*n* = 199)
Positioning	5 (5.3)	5 (14.3)	9 (13.0)	19 (9.5)
Surgical procedure	16 (16.8)	2 (5.7)	21 (30.4)	39 (19.6)
Anatomic manipulation	3 (3.2)	0 (0.0)	8 (11.6)	11 (5.5)
Anesthetic procedure	17 (17.9)	4 (11.4)	11 (15.9)	32 (16.1)
Other	11 (11.6)	2 (5.7)	5 (7.2)	18 (9.0)

Data are presented as numbers (percentages).

## 4. Discussion

The present study clearly demonstrated that ETT cuff pressure changed outside the optimal range during anesthesia in otorhinolaryngologic surgery, and 96% of patients were exposed to inadequate cuff pressure during anesthesia. Diverse factors were found to cause cuff pressure changes, and surgical procedures of the head and neck region had a high risk of altering cuff pressure.

The importance of detecting abnormal ETT cuff pressure in operating rooms and intensive care units has been reported by previous researchers (10, 16). However, this is the first study to observe changes in ETT cuff pressure throughout the anesthesia period in otorhinolaryngologic surgery. In our study, the mean TTR was 79.7, and 34.2% of the patients had inadequate cuff pressure for more than 20% of the total anesthesia time. Cuff pressure may be periodically checked with a cuff pressure manometer or an intermittent cuff-pressure system as needed (17, 18). However, in case of position change and use of surgical instrument around the airway during the surgery as this research, continuously monitoring cuff pressure is needed.

Kwon et al. (19) reported a lower incidence of ETT cuff pressure abnormalities during laparoscopic cholecystectomy, at 25%. Similarly, Park et al. (2) showed that the cuff pressure of 25% of patients exceeded 30 cmH_2_O in patients who underwent thyroidectomy. This is contrary to our findings, in which a high incidence of 96% of patients experienced inadequate cuff pressure. The reason for this difference is thought to be that most of the previous studies focused only on one situational change, such as pneumoperitoneum or head position change, and only observed cuff pressure changes intermittently twice before and after the procedure. In our study, several factors that could occur during surgery were considered simultaneously while continuously monitoring cuff pressure. In addition, it is thought that a high incidence rate was observed because our study targeted otorhinolaryngologic surgery, in which both surgeons and anesthesiologists operate in the airways.

In this study, TTR was the lowest, with a mean value of 69%, in head and neck surgeries. This indicates that the cuff pressure was least maintained in the optimal range in head and neck surgeries, possibly because head and neck surgery requires a variety of patient postures compared with otologic and nasal surgeries, manipulating of surgical instruments near the airway, and pulling and pressing tissues around the airway. In addition, cuff pressure increased as the head was rotated in otologic or nasal surgery. Our findings are similar to those of a previous report by Okgun Alcan et al. (20) which demonstrated that postural changes in head extension and neck rotation increased cuff pressure among patients in intensive care units. Moreover, neck flexion is especially vulnerable to increased cuff pressure (21, 22). Since a raise in cuff pressure is highly associated with postoperative airway complications, such as stridor and sore throat (4, 17, 23), it is appropriate to minimize changes in the patient’s neck posture. Nevertheless, movement of the head and neck is necessary to facilitate surgical access, and a typical example is otorhinolaryngologic surgery, as well as thyroid surgery and cervical spine surgery (24). Therefore, we suggest that continuous monitoring of the ETT cuff pressure should be implemented, especially in otorhinolaryngologic surgeries requiring position changes near the airway.

In addition, we observed that not only the patient’s posture but also various factors, such as nasal gauze packing, manipulation of surgical instruments, or patient tissue pulling, had a significant effect on cuff pressure. Therefore, during anesthesia, close attention should be given to various stimuli applied to the patient, and efforts should be made to prevent increases in cuff pressure.

In our study, we observed a decrease in the cuff pressure when an instrument, such as a suspension laryngoscope placed in the mouth, was removed. The underinflation of the ETT cuff can cause leakage of inhalation anesthetics and critical hypoventilation. Moreover, it is one of the risk factors for ventilator-associated pneumonia through the aspiration of subglottic sputum into the lower respiratory tract (25). The risk of silent aspiration increases if the cuff is not objectively adjusted using a manometer and evaluated by a subjective method, such as pilot balloon palpation or auscultation (10). Therefore, it is necessary to keep a close eye on the time point of surgical instrument removal and accurately monitor cuff pressure.

Moreover, duration of surgical procedure is an important factor for monitoring cuff pressure. In this study, mean of anesthesia time was 148.1 min. According to Kako et al.’s (15) research, there was more significant variations in the intracuff pressure during prolonged surgical procedures. In the longer duration of intubation more than 3 h, more complications such as sore throat and hoarseness occurred because of the high cuff pressure (26). The present study had certain limitations. First, we did not investigate the postoperative complications related to abnormal cuff pressure. We focused on detecting cuff overinflation and underinflation by continuously monitoring cuff pressure in otorhinolaryngologic surgery and found various factors. Second, our research is focused on the patients undergoing otorhinolaryngologic procedure. However, the general message on monitoring cuff pressure can be applied to all type of surgery, especially long-lasting procedure. Finally, we tried to exclude technical problems, such as dropping equipment during continuous monitoring, short temporary drops, or high outliers in cuff pressure at some seconds, by using smoothing methods. In addition to the smoothing method, there may be other more suitable methods.

## Conclusion

In conclusion, the ETT cuff pressure increased or decreased outside the appropriate range in most of the patients undergoing otorhinolaryngologic surgery. Various factors inducing improper cuff pressure were also found. Therefore, we suggest close and continuous monitoring of cuff pressure during anesthesia for otorhinolaryngologic surgery to prevent critical overinflation and underinflation for a significant period, especially for the long-lasting surgery and in case that the position is changed.

## Data availability statement

The original contributions presented in this study are included in the article/Supplementary material, further inquiries can be directed to the HK, jjollong@gmail.com.

## Ethics statement

This study was approved by the Yonsei University’s Institutional Review Board (IRB # 4-2019-0100), and written informed consent was obtained from all the individuals participating in the trial. The patients/participants provided their written informed consent to participate in this study.

## Author contributions

SP, YK, and HK: conception and design of the work, acquisition, analysis, drafted, and revised it. SP and HK: interpretation of data and substantively revised the work. All authors reviewed and commented on drafts of the manuscript.

## Footnotes

1
http://www.R-project.org

## References

[1] Letvin A Kremer P Silver P Samih N Reed-Watts P Kollef M. Frequent versus infrequent monitoring of endotracheal tube cuff pressures. *Respir Care.* (2018) 63:495. 10.4187/respcare.05926 29382793

[2] Park J Lee H Lee S Kim J. Changes in tapered endotracheal tube cuff pressure after changing position to hyperextension of neck: a randomized clinical trial. *Medicine.* (2021) 100:e26633. 10.1097/MD.0000000000026633 34398020 PMC8294867

[3] Dobrin P Canfield T. Cuffed endotracheal tubes: mucosal pressures and tracheal wall blood flow. *Am J Surg.* (1977) 133:562–8. 10.1016/0002-9610(77)90008-3 860779

[4] Shaikh F Janaapureddy Y Mohanty S Reddy P Sachane K Dekate P Utility of endotracheal tube cuff pressure monitoring in mechanically ventilated (MV) children in preventing post-extubation stridor (PES). *Indian J Crit Care Med.* (2021) 25:181–4. 10.5005/jp-journals-10071-23737 33707897 PMC7922465

[5] Grant T. Do current methods for endotracheal tube cuff inflation create pressures above the recommended range? A review of the evidence. *J Perioper Pract.* (2013) 23:198–201. 10.1177/175045891302300904 24245362

[6] Viswambharan B Kumari M Krishnan G Ramamoorthy L. Under or overpressure: an audit of endotracheal cuff pressure monitoring at the tertiary care center. *Acute Crit Care.* (2021) 36:374–9. 10.4266/acc.2021.00024 34736298 PMC8907457

[7] Rokamp K Secher N Møller A Nielsen H. Tracheal tube and laryngeal mask cuff pressure during anaesthesia-mandatory monitoring is in need. *BMC Anesthesiol.* (2010) 10:20. 10.1186/1471-2253-10-20 21129183 PMC3016349

[8] Lee J Reynolds H Pelecanos A van Zundert A. Bi-national survey of intraoperative cuff pressure monitoring of endotracheal tubes and supraglottic airway devices in operating theatres. *Anaesth Intensive Care.* (2019) 47:378–84. 10.1177/0310057X19850581 31280594

[9] Rosero E Ozayar E Eslava-Schmalbach J Minhajuddin A Joshi G. Effects of increasing airway pressures on the pressure of the endotracheal tube cuff during pelvic laparoscopic surgery. *Anesth Analg.* (2018) 127:120–5. 10.1213/ANE.0000000000002657 29189283

[10] Hockey C Van Zundert A Paratz J. Does objective measurement of tracheal tube cuff pressures minimise adverse effects and maintain accurate cuff pressures? A systematic review and meta-analysis. *Anaesth Intensive Care.* (2016) 44:560–70. 10.1177/0310057x1604400503 27608338

[11] Ganason N Sivanaser V Liu C Maaya M Ooi J. Post-operative sore throat: comparing the monitored endotracheal tube cuff pressure and pilot balloon palpation methods. *Malays J Med Sci.* (2019) 26:132–8. 10.21315/mjms2019.26.5.12 31728125 PMC6839667

[12] Brimacombe J Keller C Giampalmo M Sparr H Berry A. Direct measurement of mucosal pressures exerted by cuff and non-cuff portions of tracheal tubes with different cuff volumes and head and neck positions. *Br J Anaesth.* (1999) 82:708–11. 10536547 10.1093/bja/82.5.708

[13] Knowlson G Bassett H. The pressures exerted on the trachea by endotracheal inflatable cuffs. *Br J Anaesth.* (1970) 42:834–7. 10.1093/bja/42.10.834 4920121

[14] Reiffel J. Time in the Therapeutic Range (TTR): an Overly Simplified Conundrum. *J Innov Card Rhythm Manag.* (2017) 8:2643–6. 10.19102/icrm.2017.080302 32494441 PMC7252837

[15] Kako H Goykhman A Ramesh A Krishna S Tobias J. Changes in intracuff pressure of a cuffed endotracheal tube during prolonged surgical procedures. *Int J Pediatr Otorhinolaryngol.* (2015) 79:76–9. 10.1016/j.ijporl.2014.11.017 25487872

[16] Sengupta P Sessler D Maglinger P Wells S Vogt A Durrani J Endotracheal tube cuff pressure in three hospitals, and the volume required to produce an appropriate cuff pressure. *BMC Anesthesiol.* (2004) 4:8. 10.1186/1471-2253-4-8 15569386 PMC535565

[17] Yildirim Z Uzunkoy A Cigdem A Ganidagli S Ozgonul A. Changes in cuff pressure of endotracheal tube during laparoscopic and open abdominal surgery. *Surg Endosc.* (2012) 26:398–401. 10.1007/s00464-011-1886-8 21909860

[18] Lorente L Lecuona M Jiménez A Lorenzo L Roca I Cabrera J Continuous endotracheal tube cuff pressure control system protects against ventilator-associated pneumonia. *Crit Care.* (2014) 18:R77. 10.1186/CC13837 24751286 PMC4057071

[19] Kwon Y Jang J Hwang S Lee J Hong S Hong S The change of endotracheal tube cuff pressure during laparoscopic surgery. *Open Med.* (2019) 14:431–6. 10.1515/med-2019-0046 31198857 PMC6555239

[20] Okgun Alcan A Yavuz van Giersbergen M Dincarslan G Hepcivici Z Kaya E Uyar M. Effect of patient position on endotracheal cuff pressure in mechanically ventilated critically ill patients. *Aust Crit Care.* (2017) 30:267–72. 10.1016/j.aucc.2016.11.006 27993545

[21] Komasawa N Mihara R Imagawa K Hattori K Minami T. Comparison of pressure changes by head and neck position between high-volume low-pressure and taper-shaped cuffs: a randomized controlled trial. *Biomed Res Int.* (2015) 2015:386080. 10.1155/2015/386080 26509152 PMC4609783

[22] Kim D Jeon B Son J Lee J Ko S Lim H. The changes of endotracheal tube cuff pressure by the position changes from supine to prone and the flexion and extension of head. *Korean J Anesthesiol.* (2015) 68:27–31. 10.4097/kjae.2015.68.1.27 25664152 PMC4318861

[23] Ryu J Han S Do S Lee J Lee S Choi E. Effect of adjusted cuff pressure of endotracheal tube during thyroidectomy on postoperative airway complications: prospective, randomized, and controlled trial. *World J Surg.* (2013) 37:786–91. 10.1007/s00268-013-1908-x 23334802

[24] Li Y Tan E Tsai Y Mandell M Huang S Chiang T A tapered cuff tracheal tube decreases the need for cuff pressure adjustment after surgical retraction during anterior cervical spine surgery: a randomized controlled. *Double-Blind Trial. Front Med.* (2022) 9:920726. 10.3389/fmed.2022.920726 35847807 PMC9276934

[25] Rouzé A Nseir S. Continuous control of tracheal cuff pressure for the prevention of ventilator-associated pneumonia in critically ill patients: where is the evidence? *Curr Opin Crit Care.* (2013) 19:440–7. 10.1097/MCC.0B013E3283636B71 23856895

[26] Liu J Zhang X Gong W Li S Wang F Fu S Correlations between controlled endotracheal tube cuff pressure and postprocedural complications: a multicenter study. *Anesth Analg.* (2010) 111:1133–7. 10.1213/ANE.0b013e3181f2ecc7 20736432

