# Supplementary material for: Pressure changes in the endotracheal tube cuff in otorhinolaryngologic surgery: a prospective observational study
Source: Front Med (Lausanne). 2023 Jun 5;10:1161566. doi: 10.3389/fmed.2023.1161566 (PMC10277466; doi:10.3389/fmed.2023.1161566)
Supplement: Supplementary file 1 [file Presentation_1.pdf]

Supplementary Figures

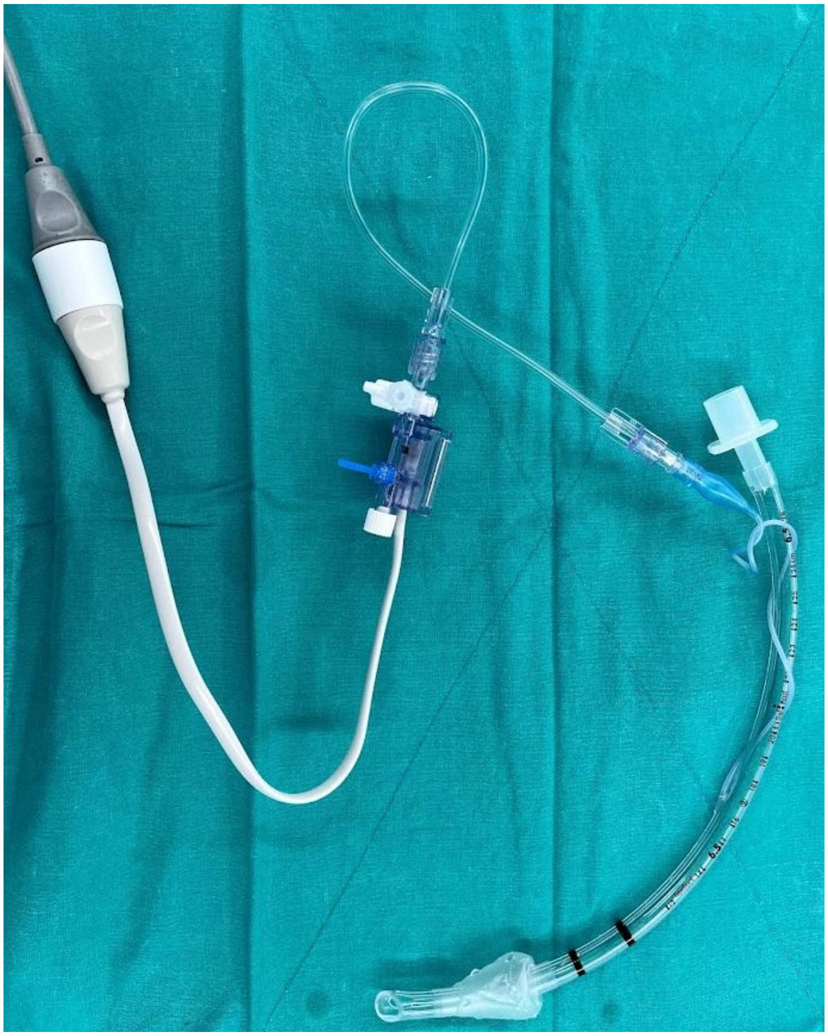

Supplementary Figure 1. A transducer for monitoring the cuff pressure of the endotracheal tube.

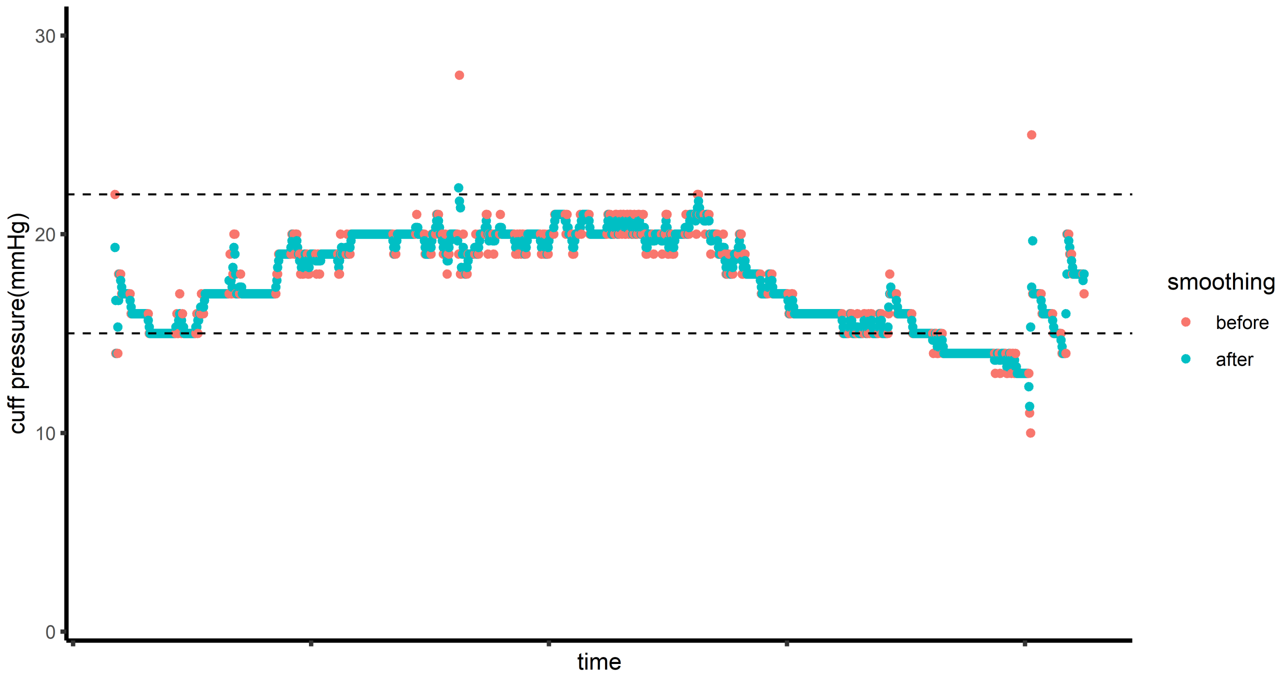

Supplementary Figure 2. An example of applying the smoothing methods before the calculation of TTR.
